# Clinical Applicability of the WHO Reporting System for Axillary Lymph Node Fine-Needle Aspiration in Invasive Breast Carcinoma

**DOI:** 10.3390/cancers18172847

**Published:** 2026-09-03

**Authors:** Mariana Canepa, Allison Chang, Stephanie L. Graff, Yihong Wang

**Affiliations:** 1 Department of Pathology and Laboratory Medicine, Rhode Island Hospital, Brown University Health, Providence, RI 02903, USA; 2 Division of Medical Oncology, Rhode Island Hospital, Brown University Health, Providence, RI 02903, USA

**Keywords:** breast carcinoma, breast cancer, axillary lymph node, cytology, FNA

## Abstract

Background: Axillary lymph node (ALN) fine-needle aspirations (FNAs) can guide the next steps in the management of patients with invasive breast carcinoma (BC). The objective is to evaluate the diagnostic performance of ALN FNA in the detection of metastatic breast carcinoma. Methods: We evaluated ALN FNAs from 2020 to 2024 to determine the risk of malignancy (ROM) of different cytologic diagnostic categories using a general WHO cytology reporting system framework (insufficient, benign, atypical, suspicious, and malignant). Results: A total of 290 axillary lymph node FNAs from patients with newly diagnosed invasive breast carcinoma were identified, of which 226 (78%) had histologic and/or adequate clinical follow-up. Cytologic diagnoses included six (2.1%) non-diagnostic, 99 (34.1%) benign, two (0.7%) atypical, seven (2.4%) suspicious for malignancy, and 176 (60.7%) malignant cases. The risk of malignancy (ROM) was 50.0% for non-diagnostic, 17.0% for benign, 100% for atypical, 85.7% for suspicious, and 99.2% for malignant diagnoses. Most false-negative benign cases were attributed to sampling limitations rather than interpretive error, with five cases showing only a single sentinel lymph node metastasis measuring <5 mm. Indeterminate diagnoses were most commonly associated with scant cellularity, acellular cell blocks, obscuring blood or fibrin, and equivocal immunohistochemistry. Excluding cases without histologic follow-up, ALN FNA demonstrated a sensitivity of 86.5%, specificity of 97.4%, positive predictive value of 98.5%, negative predictive value of 79.2%, and overall accuracy of 90.3% for the detection of metastatic breast carcinoma (OR = 243.2, Fisher’s exact test, *p* < 0.0001). Conclusion: ALN FNA is a highly reliable method for confirming axillary lymph node metastasis in invasive breast carcinoma when malignant cells are identified, with a ROM of 99% for the malignant category. The suspicious category was also strongly associated with malignancy (ROM 86%). However, the 17% ROM observed in the benign category indicates that a negative FNA does not reliably exclude metastatic disease, and surgical nodal staging may still be warranted in clinically or radiologically suspicious cases. These findings support the clinical applicability of the WHO System for Reporting Lymph Node Cytopathology and provide breast carcinoma-specific ROM estimates for this clinical setting.

## Simple Summary

A standard reporting system framework including unsatisfactory, negative, atypical, suspicious for malignancy and malignant categories can be applied to axillary fine-needle aspiration (FNA) results in patients with breast carcinoma. ALN FNA is highly specific and has excellent positive predictive value for confirming metastatic breast carcinoma, but negative results should be interpreted with caution given the 17% risk of malignancy (ROM) in the benign category. The “suspicious” category, although with limited cases, carries a ROM of 86%, strongly indicating the presence of tumor. Uncertainty in FNA diagnoses (atypical/suspicious) is attributed to factors like scant cellularity, the presence of lobular carcinoma, and obscuring artifacts, representing potential diagnostic pitfalls.

## 1. Introduction

Breast carcinoma is the most common malignancy among women, excluding nonmelanoma skin cancers, and its incidence continues to increase [1]. The prevalence of lymph node metastases in the United States increases progressively with tumor size, from 10.8% for tumors measuring 1–10 mm to 61.8% for tumors measuring 51–60 mm, according to a study based on Surveillance, Epidemiology, and End Results (SEER) registry data from 1990 to 2014 [2].

Preoperative axillary lymph node (ALN) assessment provides preliminary staging information to guide management decisions for patients with invasive breast carcinoma (BC). It can also help identify patients with nodal metastasis who may proceed to lymph node dissection without the need for intraoperative sentinel node assessment [3,4]. Preoperative ALN assessment is typically recommended in patients with clinically palpable ALNs, locally advanced breast carcinoma (including large primary tumors, skin involvement, or inflammatory breast carcinoma), or suspicious imaging findings. Ultrasound features suggestive of nodal metastasis include cortical thickening, loss or effacement of the fatty hilum, rounded nodal morphology, asymmetric cortical enlargement, and increased lymph node size [5,6,7]. Core biopsy may provide greater sensitivity than fine-needle aspiration (FNA) for detecting ALN metastasis (88% vs. 72.5%) [8]. However, FNA offers several advantages, including lower cost, minimal invasiveness, and rapid results [3,9].

Studies evaluating the diagnostic performance of ALN FNA in patients with BC are limited [10,11,12,13]. The predictive values associated with the atypical and suspicious for malignancy diagnostic categories remain poorly defined in this specific clinical setting. Therefore, the clinical significance of these cytologic categories in relation to subsequent pathologic outcomes remains uncertain.

The increasing application of FNA in lymph node pathology has led to the development of a standardized reporting system, formalized in the World Health Organization (WHO) Reporting System for Lymph Node, Spleen and Thymus Cytopathology (WHO System). The WHO System was established through a joint project of the WHO, the International Agency for Research on Cancer (IARC), and the International Academy of Cytology (IAC) and comprises five diagnostic categories: inadequate/insufficient/non-diagnostic, benign, atypical, suspicious for malignancy, and malignant [14,15,16]. However, the application of the WHO System to ALN FNA specifically for the evaluation of metastatic BC has not been studied.

The WHO International Cytopathology Reporting Systems were developed to standardize reporting, improve communication between pathologists and clinicians, and provide an estimated risk of malignancy (ROM) for each diagnostic category. Knowledge of the ROM associated with each category helps guide clinical management and facilitates multidisciplinary decision-making [17,18,19,20,21,22]. However, intermediate categories such as atypical and suspicious for malignancy remain challenging to translate into clinical management. The “atypical” category is intended to maintain a high negative predictive value (NPV) for the “benign” category, whereas the “suspicious for malignancy” category is designed to preserve a high positive predictive value (PPV) for the “malignant” category. Although necessary to maintain the diagnostic performance of the benign and malignant categories, these intermediate diagnoses may create uncertainty regarding subsequent management for surgeons, oncologists, and other members of the multidisciplinary team, often requiring correlation with clinical and imaging findings, repeat sampling, or multidisciplinary discussion before definitive management decisions can be made [17,18,19,20,21,22].

Although the WHO System for Reporting Lymph Node Cytopathology provides a valuable framework for standardized reporting and risk stratification, its category-specific ROMs may not be directly applicable to ALN FNAs performed for staging invasive breast carcinoma. The WHO System was developed using heterogeneous populations encompassing reactive and infectious lymphadenopathies, lymphoproliferative disorders, and metastatic malignancies [14,15]. Published WHO estimates demonstrate a progressive increase in pooled ROM from 5% for benign and 65% for atypical diagnoses to 93% for suspicious for malignancy and 100% for malignant diagnoses, with a pooled ROM of 34% for non-diagnostic specimens [14]. In contrast, ALN FNA in patients with invasive breast carcinoma is performed in a highly selected clinical setting in which metastatic disease is the primary diagnostic consideration. Given the higher pretest probability of malignancy, the ROM of individual WHO categories may therefore differ from estimates derived from unselected lymph node populations. Defining and comparing category-specific ROMs in this disease-specific population may therefore provide more clinically relevant risk estimates for patient management.

The aim of this study is to evaluate the diagnostic performance of ALN FNA in patients with invasive breast carcinoma and to assess the clinical applicability of the WHO System for Reporting Lymph Node Cytopathology in this setting.

## 2. Design

Upon approval by the institutional review board, a retrospective search of the pathology database was performed to identify ALN FNA specimens obtained between January 2020 and February 2024. Cases were identified using the search terms “axilla” and “axillary” in the final cytology diagnosis. Cases with corresponding histologic follow-up, including core needle biopsy, sentinel lymph node biopsy, axillary lymph node dissection (ALND), and/or adequate clinical follow-up, were included. Cases of lymphoma, melanoma, and metastatic malignancies of non-breast origin were excluded. All cytology slides were retrospectively reviewed by a single cytopathologist and categorized according to the WHO System for Reporting Lymph Node Cytopathology. The reviewer was not blinded to the original cytologic diagnosis or subsequent histologic and clinical follow-up. Retrospective review did not result in reclassification of any case, as all original cytologic diagnoses were concordant with the corresponding WHO diagnostic categories. Because the review was performed by a single cytopathologist, interobserver agreement was not assessed.

Cytology findings were correlated with subsequent surgical pathology results obtained from the pathology information system and clinical follow-up information retrieved from the electronic medical record. Recorded clinicopathologic variables included patient age and sex, cytologic diagnosis, type of histologic follow-up, breast carcinoma subtype, Nottingham grade, estrogen receptor (ER), progesterone receptor (PR), and HER2 status, pathologic stage, receipt of neoadjuvant chemotherapy, presence of distant metastases, number of sentinel lymph nodes examined, size of the largest nodal metastasis, and whether axillary lymph node dissection (ALND) was performed.

All FNAs were performed under ultrasound guidance by interventional radiologists using 22- to 25-gauge needles. Aspirated material was collected in CytoLyt^®^ solution (Hologic, Inc., Marlborough, MA, USA), processed using the ThinPrep^®^ system (Hologic, Inc., Marlborough, MA, USA), and stained with the Papanicolaou method. Cell blocks were prepared from residual material and processed as formalin-fixed paraffin-embedded specimens according to standard laboratory protocols. Rapid on-site evaluation (ROSE) was not performed. All cytology slides were retrospectively reviewed and categorized according to the WHO System for Reporting Lymph Node Cytopathology. Immunohistochemical studies were performed on selected cell block sections at the discretion of the pathologist to clarify indeterminate findings or confirm breast origin. Antibodies included cytokeratin cocktail (AE1/AE3 and CAM5.2; Dako, Glostrup, Denmark and Biogenex, Fremont, CA, USA), GATA3 (Monoclonal; L50-823; Biocare Medical, Pacheco, CA, USA), and cytokeratin 7 (M; OV- TL12/30; DAKO, Glostrup, Denmark). Briefly, formalin-fixed paraffin-embedded cell block sections underwent antigen retrieval followed by incubation with primary antibodies and visualization using EnVision Flex, EnVision + Dual Link System (Dako, Glostrup, Denmark), UltraView Universal DAB Detection Kit (Ventana Medical Systems, Tucson, AZ, USA), or standard streptavidin-based detection systems with diaminobenzidine (DAB) or NovaRED chromogens, according to laboratory protocols.

In cases with a positive ALN FNA and a known ipsilateral breast carcinoma, metastatic breast carcinoma was presumed unless the clinical history or imaging findings raised concern for an alternative primary malignancy, in which case additional immunohistochemistry was performed to establish the site of origin. Cases determined to represent metastatic malignancies of non-breast origin were excluded from the study.

Cases were retrospectively classified according to the WHO System for Reporting Lymph Node Cytopathology as non-diagnostic, benign, atypical, suspicious for malignancy, or malignant. The benign category included specimens showing unequivocal lymphoid tissue and no evidence of metastatic carcinoma. The atypical category was reserved for cases with limited atypical epithelial cells or other features insufficient for a definitive diagnosis. The suspicious for malignancy category included cases with cytomorphologic features suggestive of metastatic carcinoma but insufficient in quality or quantity to establish a malignant diagnosis. The malignant category included cases with unequivocal cytomorphologic evidence of metastatic carcinoma.

**Figure 1:**
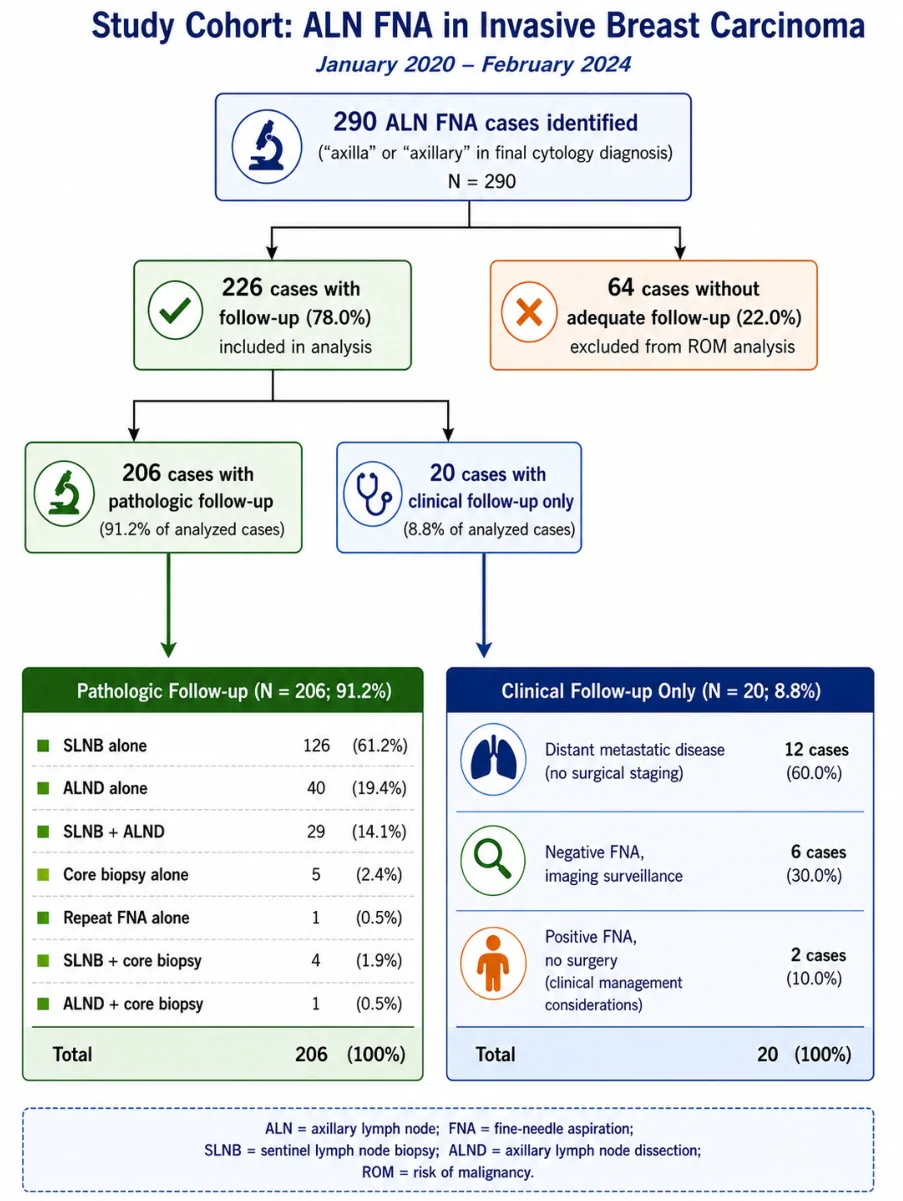
Study cohort and follow-up of axillary lymph node fine-needle aspiration cases. Flow diagram showing selection and follow-up of ALN FNA cases from patients with invasive breast carcinoma identified between January 2020 and February 2024. Of 290 cases identified, 226 (78.0%) had adequate pathologic and/or clinical follow-up and were included in the outcome analysis, whereas 64 (22.0%) without adequate follow-up were excluded from ROM calculations. Among the 226 analyzed cases, 206 (91.2%) had pathologic follow-up and 20 (8.8%) were evaluated by clinical follow-up alone. The figure summarizes the types of pathologic and clinical follow-up used in the study. ALN, axillary lymph node; FNA, fine-needle aspiration; SLNB, sentinel lymph node biopsy; ALND, axillary lymph node dissection; ROM, risk of malignancy.

Of the 290 identified cases, 226 (78.0%) had adequate pathologic and/or clinical follow-up and were included in the outcome analysis, whereas 64 (22.0%) were excluded from ROM calculations because of insufficient follow-up (Figure 1). Among the 226 included cases, 206 had additional pathologic evaluation and 20 (8.8%) were evaluated by clinical follow-up alone. Clinical follow-up was used when surgical nodal staging was not performed and included patients with distant metastatic disease, elderly patients with negative ALN FNA who remained free of radiologic evidence of axillary disease, and elderly patients with positive ALN FNA who did not undergo surgery based on clinical management decisions.

### Statistical Analysis

Cases without histologic follow-up were excluded from diagnostic performance analyses. Cytology diagnoses categorized as “suspicious for malignancy” and “malignant” were considered positive test results, whereas diagnoses categorized as “insufficient,” “benign,” and “atypical” were considered negative test results. Diagnostic performance metrics, including sensitivity, specificity, positive predictive value (PPV), negative predictive value (NPV), and overall accuracy, were calculated using histologic follow-up as the reference standard. The association between cytologic diagnosis and histologic outcome was evaluated using Fisher exact test. Odds ratios (ORs) with corresponding 95% confidence intervals (CIs) were calculated from 2 × 2 contingency tables. Statistical analyses were performed using Python (version 3.x) with the SciPy and Statsmodels libraries (SciPy, https://scipy.org/; Statsmodels, https://www.statsmodels.org/; accessed 6 July 2026). A two-sided *p*-value < 0.05 was considered statistically significant.

## 3. Results

A total of 290 ALN FNAs from patients with newly diagnosed BC were identified. Cytologic diagnoses included six insufficient (2.1%), 99 benign (34.1%), two atypical (0.7%), seven suspicious for malignancy (2.4%), and 176 malignant (60.7%).

Of the 290 cases, 226 (78%) had adequate pathologic and/or clinical follow-up (Figure 1). Follow-up consisted of SLNB alone in 126 cases (55.8%), ALND alone in 40 cases (17.7%), combined SLNB and ALND in 29 cases (12.8%), core lymph node biopsy alone in five cases (2.2%), repeat FNA alone in one case (0.4%), SLNB with core lymph node biopsy in four cases (1.8%), and ALND with core lymph node biopsy in one case (0.4%). The remaining 20 cases (8.8%) were evaluated by clinical follow-up alone. These included 12 patients with distant metastatic disease who did not undergo surgical nodal staging, six elderly patients with negative ALN FNA results who were managed conservatively and remained free of radiologic evidence of axillary disease on follow-up imaging, and two elderly patients with positive ALN FNA results who did not undergo surgery based on age and clinical management decisions.

**Figure 2:**
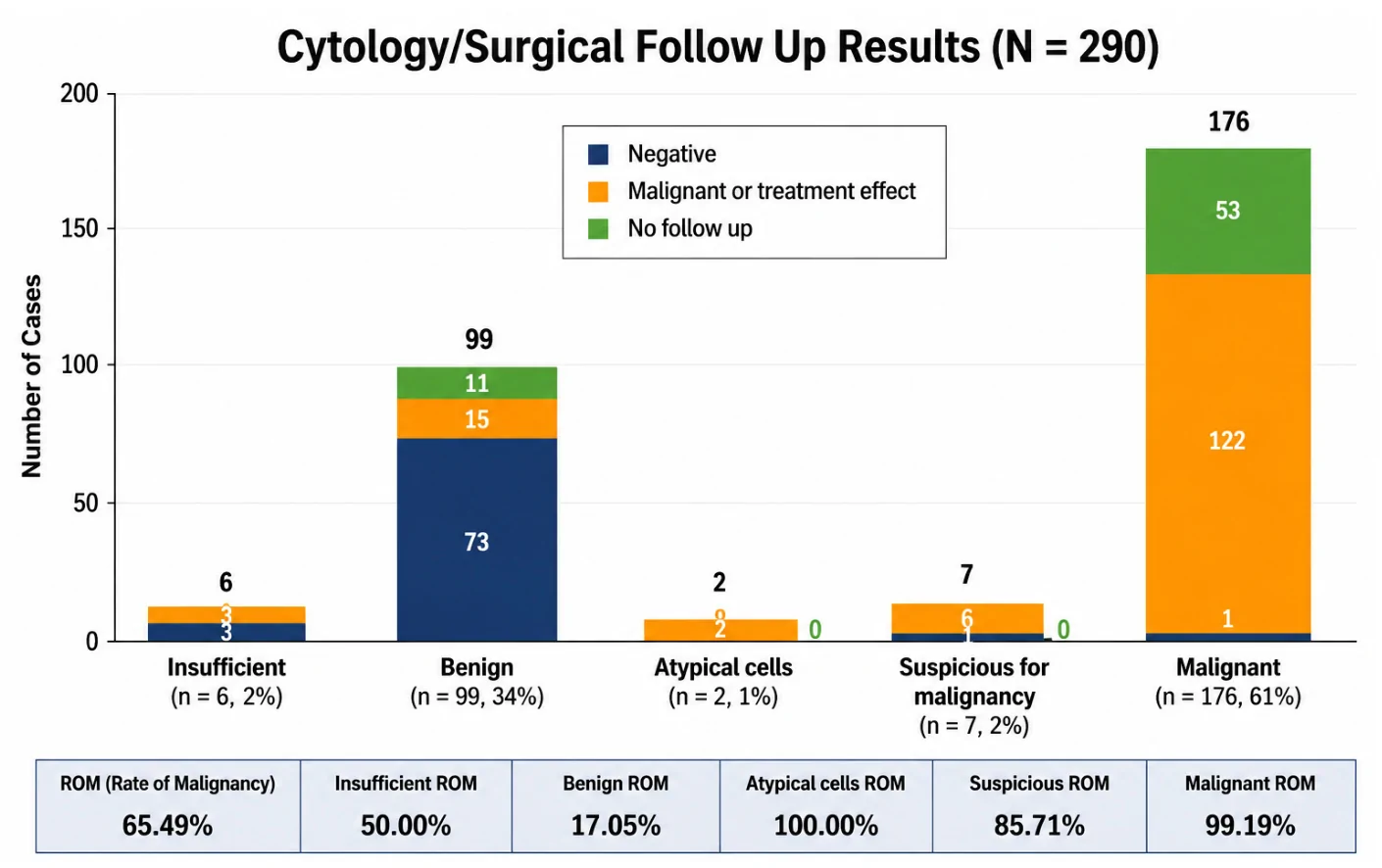
Cytologic diagnostic categories, follow-up outcomes, and risk of malignancy for axillary lymph node fine-needle aspiration. Distribution of the 290 ALN FNA cases according to cytologic diagnostic category and subsequent outcome. Bars show cases with negative follow-up, metastatic carcinoma or treatment effect, and no available follow-up. Category-specific risk of malignancy (ROM) was calculated among cases with adequate follow-up. ROM was 50.0% for non-diagnostic/insufficient, 17.0% for benign, 100% for atypical, 85.7% for suspicious for malignancy, and 99.2% for malignant cytology. Cases showing lymph node treatment effect following neoadjuvant chemotherapy were considered positive outcomes. ALN, axillary lymph node; FNA, fine-needle aspiration; ROM, risk of malignancy.

Six non-diagnostic cytology cases had pathologic follow-up, with metastatic carcinoma identified in three (ROM, 50.0%) (Figure 2). The non-diagnostic specimens ranged from acellular samples to those containing only rare lymphocytes.

Among 88 benign FNA cases with follow-up, 15 were positive on subsequent lymph node excision (ROM, 17.0%) (Figure 2). Review of these false-negative cases did not identify tumor cells on the cytology slides, although three cases showed scant cellularity. Immunohistochemistry was performed on cell block sections in five cases. Among the 15 benign FNAs with malignant follow-up, five had a single sentinel lymph node containing a metastasis measuring less than 5 mm. In the remaining cases, the metastatic deposits identified on subsequent excision were generally of sufficient size to suggest that the false-negative FNA resulted from failure to sample the metastatic focus rather than failure to recognize tumor cells cytologically.

**Figure 3:**
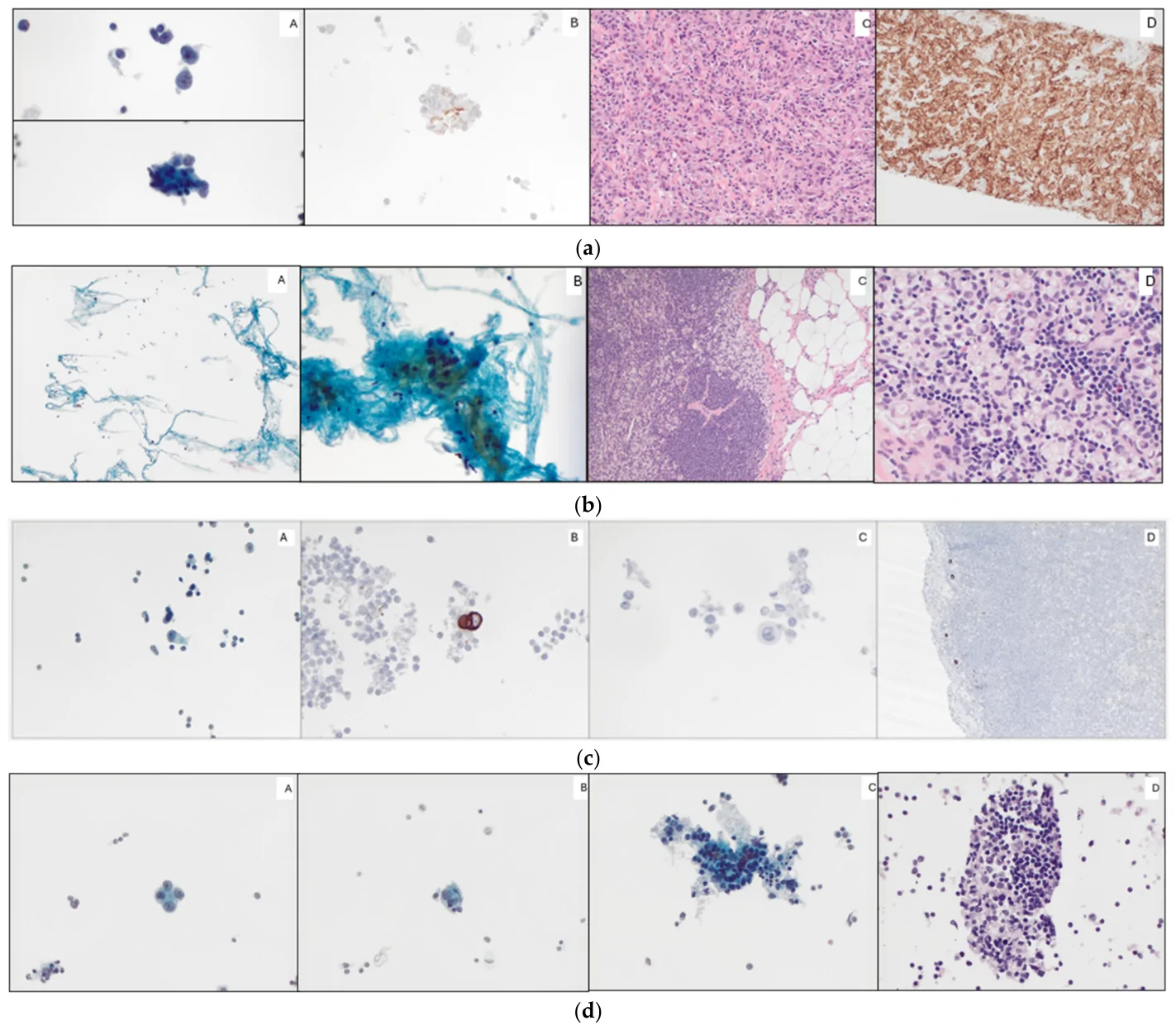
(**a**). A. Atypical ALN FNA showing scattered individual atypical cells with scant cytoplasm morphologically resembling lymphocytes (A, ThinPrep^®^ Pap 400×). B. Rare atypical cells on the cell block with negative cytokeratin7 (400×). Subsequent core biopsy demonstrated poorly differentiated triple-negative breast carcinoma with singly infiltrating tumor cells (C, H&E 200×) strongly positive for cytokeratin cocktail (D, 100×). (**b**). Atypical ALN FNA showing a single group of atypical cells entrapped in fibrin (B, ThinPrep^®^ Pap 400×) in a peripheral blood background (A Pap 100×); the cell block was acellular. Concurrent breast biopsy demonstrated invasive carcinoma with mixed ductal and lobular features. C. Follow-up sentinel lymph node with metastatic subcapsular carcinoma (mixed ductal and lobular on concurrent breast biopsy) (H&E 100×). D. High-power view of the carcinoma cells (H&E 400×). (**c**). Suspicious ALN FNA from a patient with classic invasive lobular carcinoma showing scant atypical cells; subsequent sentinel lymph node biopsy demonstrated isolated tumor cells. A. ThinPrep^®^ with suspicious for malignancy diagnosis: a single atypical cell with eccentric nucleus and a cytoplasmic vacuole (Pap 400×). B. Rare positive cytokeratin cocktail staining on the cell block (400×). C. Negative GATA-3 staining of the cell block (400×). D. Isolated tumor cells on the sentinel node resection highlighted by cytokeratin cocktail (200×). (**d**). Suspicious ALN FNA with atypical cells suggestive of metastatic carcinoma. Following neoadjuvant chemotherapy, subsequent SLNB performed 5.5 months later was negative without identifiable treatment effect. A. and B. ThinPrep with scattered small groups suspicious for carcinoma (Pap 400×). C. The cells look different from the dendritic cells in these lymphocytic aggregates (Pap 400×). D. Cell block with what appears to be normal lymph node fragments (H&E 400×). No immunostaining was performed in this case.

Both atypical cases were positive on surgical follow-up (ROM, 100%) (Figure 2). One case showed scattered individual atypical cells with scant cytoplasm that morphologically resembled lymphocytes; cytokeratin 7 immunohistochemistry was equivocal. Subsequent ALN core biopsy demonstrated poorly differentiated carcinoma composed of singly infiltrating cells that were strongly positive for cytokeratin cocktail (Figure 3a). The other atypical case showed a peripheral blood background with a single group of atypical cells entrapped in fibrin and an acellular cell block. The concurrent breast biopsy demonstrated invasive carcinoma with mixed ductal and lobular features (Figure 3b).

For the suspicious-for-malignancy category considered independently, six of seven cases were confirmed as malignant, corresponding to a positive predictive value of 85.7% (95% CI, 48.7–97.4%) (Figure 2). An independent negative predictive value cannot be meaningfully calculated for the suspicious category because it represents a single positive diagnostic tier rather than a binary diagnostic test. All cases contained fewer than five scattered atypical cells or fewer than three groups of cells that were either cytologically bland but clearly epithelial or highly atypical. Cell blocks were acellular in four of seven cases, and immunohistochemistry findings were equivocal in three of seven, limiting definitive classification as metastatic carcinoma. One case involved classic invasive lobular carcinoma, with only isolated tumor cells identified in the sentinel lymph node (Figure 3c).

The single suspicious case with negative follow-up was reviewed both internally and externally, with consensus agreement on the cytologic interpretation (Figure 3d). Following neoadjuvant chemotherapy, no treatment effect was identified in the SLN biopsy performed at an outside institution 5.5 months after the ALN FNA; the surgical slides were not available for review.

**Figure 4:**
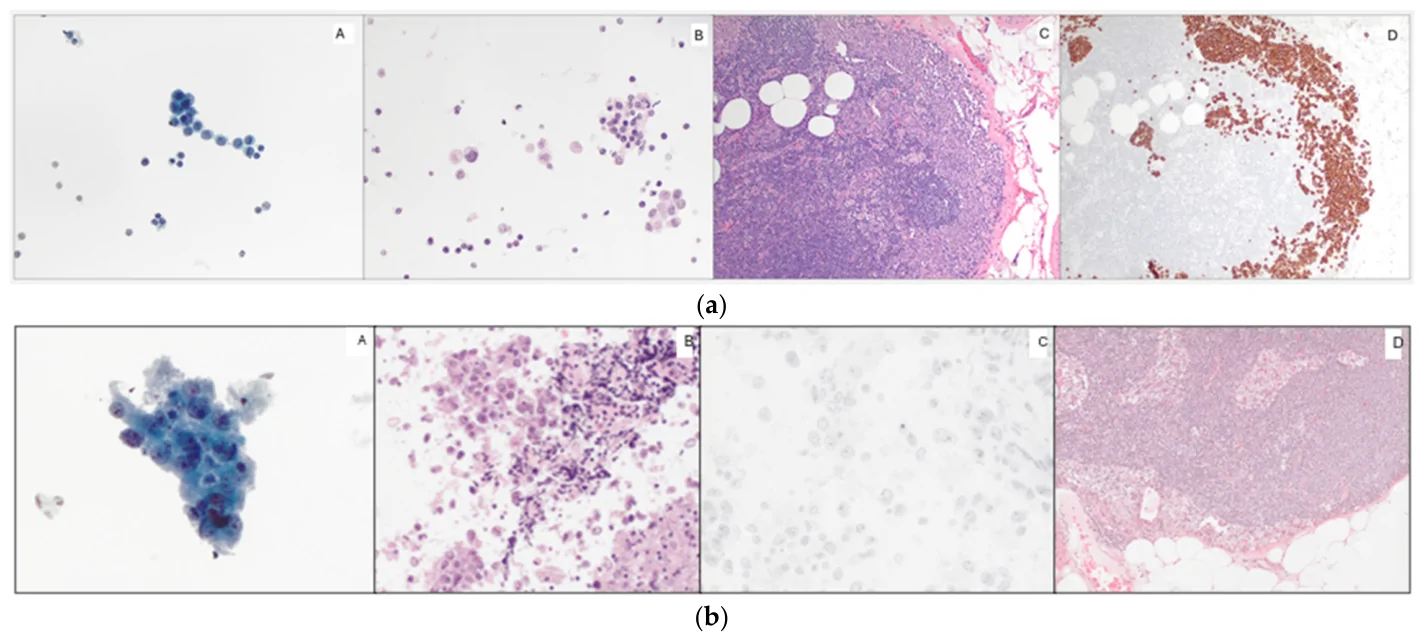
Cytologic and histologic findings in malignant ALN FNA cases. (**a**) Malignant ALN FNA with concordant histologic follow-up. A. ThinPrep^®^ preparation showing malignant cells arranged in linear groups (Papanicolaou stain, ×400). B. Cell block showing similar malignant cells (H&E, ×400). C. Subsequent lymph node dissection demonstrating metastatic invasive lobular carcinoma (H&E, ×200). D. Cytokeratin cocktail highlighting the metastatic tumor cells (×200). (**b**) Malignant ALN FNA with discordant follow-up. A. ThinPrep^®^ preparation showing groups of malignant cells (Papanicolaou stain, ×400). B. Cell block showing malignant cells with background necrosis (H&E, ×400). C. GATA3 immunohistochemical stain negative in the tumor cells (×400). D. Follow-up sentinel lymph node biopsy performed 6.5 months after the FNA, showing no metastatic carcinoma or histologic evidence of treatment effect (H&E, ×100). ALN, axillary lymph node; FNA, fine-needle aspiration.

Of 123 malignant ALN FNA cases with follow-up, 122 were considered true-positive for metastatic carcinoma (ROM/PPV, 99.2%, 122/123; 95% CI, 95.5–99.9%) (Figure 2). Follow-up consisted of positive ALNs in 69 cases, negative lymph nodes with treatment effect in 38 cases, and distant metastatic disease confirmed by biopsy or radiologic assessment in 15 cases (Figure 4a). The single malignant case with negative follow-up remained strongly suspicious for malignancy on consensus cytologic review, despite negative GATA3 staining on the cell block. SLNB performed 6 months after the FNA and following NACT demonstrated benign lymph nodes without identifiable treatment effect (Figure 4b).

Fifty-three cases diagnosed as malignant on ALN FNA lacked corresponding surgical follow-up and were excluded from ROM calculations. These cases primarily included patients treated at outside institutions, elderly or medically frail patients managed conservatively without surgical intervention, patients receiving neoadjuvant or systemic therapy without available surgical pathology at the time of review, and a small number lost to follow-up.

Among the 130 patients with malignant or suspicious ALN FNA diagnoses and available follow-up, 65 (50.0%) subsequently underwent ALND and 71 (54.6%) underwent SLNB. Forty-one patients (31.5%) underwent ALND without preceding SLNB, whereas 24 (18.5%) underwent both procedures. Among malignant FNA cases specifically, 61 of 123 underwent ALND and 68 underwent SLNB; among the seven suspicious cases, four underwent ALND and three underwent SLNB.

The ROM was 50.0% (3/6; 95% CI, 11.8–88.2%) for the non-diagnostic category, 100% (2/2; 95% CI, 15.8–100%) for the atypical category, and 85.7% (6/7; 95% CI, 42.1–99.6%) for the suspicious-for-malignancy category. The wide confidence intervals reflect the small number of cases within these categories and the resulting imprecision of the ROM estimates (Table 1).

### 3.1. Demographics

All patients were female except for one male. Age ranged from 31 to 100 years, with a mean of 64.4 years. Among the 226 cases with follow-up, the most common histologic subtype was invasive ductal carcinoma (171 cases, 75.7%), followed by invasive lobular carcinoma (22 cases, 9.7%) and mixed ductal and lobular carcinoma (18 cases, 8.0%). The remaining 15 cases (6.6%) comprised other histologic subtypes, including poorly differentiated carcinoma, mucinous carcinoma, and one combined invasive ductal and small cell carcinoma. Of the 226 patients, 127 (56.2%) received neoadjuvant chemotherapy prior to surgical evaluation. Regarding receptor status, 71.2% (161/226) had HR-positive/HER2-negative breast carcinoma, 21.7% (49/226) had triple-negative disease, 4.9% (11/226) had HR-positive/HER2-positive tumors, and 2.2% (5/226) had HR-negative/HER2-positive tumors.

In an exploratory analysis by histologic subtype, invasive lobular carcinoma showed a lower proportion of malignant cytologic diagnoses than invasive ductal carcinoma (39.1% vs. 60.8%) and a higher proportion of benign diagnoses (52.2% vs. 33.8%). Suspicious diagnoses occurred in 4.3% of ILC and 2.9% of IDC cases. These findings suggest a trend toward less definitive cytologic classification in ILC, consistent with the recognized difficulty of detecting discohesive lobular carcinoma cells; however, the small number of ILC cases limits definitive conclusions (Table 2). ALN FNA sensitivity was 89.5% (94/105) for invasive ductal carcinoma, compared with 64.3% (9/14) for invasive lobular carcinoma and 86.7% (13/15) for carcinomas with mixed ductal and lobular features. Corresponding false-negative rates were 10.5%, 35.7%, and 13.3%, respectively. The difference in sensitivity between IDC and ILC was statistically significant (Fisher’s exact test, *p* = 0.022), whereas differences between IDC and mixed carcinoma (*p* = 0.666) and ILC and mixed carcinoma (*p* = 0.215) were not significant. Given the small number of ILC and mixed cases, these findings should be considered exploratory (Table 3).

### 3.2. Accuracy of ALN FNA

Excluding cases without histologic follow-up, ALN FNA demonstrated a sensitivity of 86.5%, specificity of 97.4%, positive predictive value of 98.5%, negative predictive value of 79.2%, and overall accuracy of 90.3% for the detection of metastatic breast carcinoma. Cytologic findings were strongly associated with histologic outcome (OR = 243.2, Fisher’s exact test *p* < 0.0001), supporting the high diagnostic performance of ALN FNA in this clinical setting.

Among the 226 patients with follow-up, 127 received NACT and 99 did not. In an exploratory subgroup analysis, ALN FNA sensitivity was 94.8% (92/97) among patients who subsequently received neoadjuvant chemotherapy and 70.6% (36/51) among patients who did not receive neoadjuvant chemotherapy, corresponding to false-negative rates of 5.2% and 29.4%, respectively (Fisher exact test, *p* < 0.0001). Because ALN FNA was performed before initiation of neoadjuvant therapy and treatment selection may have been influenced by the pretreatment nodal findings, these differences should be interpreted cautiously and should not be considered evidence that neoadjuvant chemotherapy alters the intrinsic diagnostic sensitivity of FNA.

## 4. Discussion

In this study, ALN FNA demonstrated high diagnostic performance for the preoperative detection of metastatic breast carcinoma, with a sensitivity of 86.5%, specificity of 97.4%, positive predictive value of 98.5%, and overall accuracy of 90.3%. The risk of malignancy (ROM) was 99.2% in the malignant category and 85.7% in the suspicious for malignancy category, supporting the value of cytologic assessment for preoperative axillary staging and surgical planning. Previous studies have reported sensitivities of approximately 80–90% and specificities approaching 100% for ALN FNA in breast carcinoma, consistent with our findings [10,11,12,13,23,24,25].

ALN status remains one of the most important prognostic factors in breast carcinoma and plays a critical role in guiding surgical management. In patients with clinically or radiologically suspicious lymph nodes, ALN FNA provides a minimally invasive method for confirming nodal metastasis before surgery. A positive ALN FNA may influence decisions regarding neoadjuvant therapy, sentinel lymph node biopsy, and axillary lymph node dissection [26,27,28,29]. Compared with surgical staging procedures, FNA is inexpensive, rapid, well tolerated, and associated with minimal morbidity. The high specificity and positive predictive value observed in our cohort support its use for preoperative confirmation of nodal metastasis in appropriately selected patients.

Preoperative confirmation of axillary metastasis may also have practical implications for surgical management. In this cohort, 41 of 130 patients (31.5%) with malignant or suspicious ALN FNA proceeded to ALND without SLNB, demonstrating that cytologic confirmation of nodal disease can, in selected patients, establish nodal involvement before surgery and permit a management pathway that does not require sentinel node staging. However, the potential to omit SLNB depends on the overall clinical setting and treatment sequence, particularly the use of neoadjuvant therapy, and the retrospective design of this study does not permit determination of how many additional SLNB procedures could safely have been avoided.

The clinical context in which ALN FNA is performed is particularly relevant when interpreting category-specific ROM. Unlike general lymph node FNA populations, patients undergoing axillary staging for known invasive breast carcinoma have a high baseline likelihood of nodal metastasis. This selection is expected to influence the post-test probability of malignancy associated with each WHO diagnostic category. Direct comparison with published WHO ROM estimates demonstrates that this difference is not uniform across diagnostic categories (Table 4). The most clinically relevant difference was observed in the benign category, for which the ROM was 17.0% in this cohort compared with a pooled WHO estimate of 5%, representing an absolute increase of 12 percentage points and a greater than threefold relative increase. The ROM was also higher for the non-diagnostic (50.0% vs. 34%) and atypical (100% vs. 65%) categories; however, these estimates should be interpreted cautiously because only six non-diagnostic and two atypical cases were included. In contrast, the suspicious (85.7% vs. 93%) and malignant (99.2% vs. 100%) categories remained close to the high ROMs reported by the WHO System. These findings suggest that the effect of the breast carcinoma-specific clinical setting is most consequential for interpretation of non-malignant and indeterminate ALN FNA results rather than for cytologically malignant specimens. In particular, the increased ROM associated with a benign ALN FNA demonstrates that the low ROM generally associated with the WHO benign category cannot be directly extrapolated to patients with invasive breast carcinoma undergoing targeted axillary evaluation. This distinction supports disease- and clinical-context-specific validation of the WHO reporting framework and represents an important clinical contribution of this study.

The principal diagnostic challenge identified in this study involved the interpretation of the non-malignant and indeterminate categories. The benign category demonstrated a ROM of 17.0%, higher than would generally be expected for a negative cytology result. Review of the false-negative cases suggested that sampling limitations, rather than interpretive error, were the principal source of discrepancy. Most false-negative cases showed no significant cytologic atypia on retrospective review, consistent with the findings of Sauer et al., who reported that false-negative ALN FNAs generally remained morphologically negative upon slide review, similarly implicating sampling limitations [30]. Furthermore, five of the 15 benign FNAs with malignant follow-up showed only a single sentinel lymph node containing a metastasis measuring less than 5 mm. Krishnamurthy et al. also found false-negative FNAs predominantly in patients with fewer than three positive sentinel lymph nodes and metastatic deposits smaller than 0.5 cm [11]. Together, these observations suggest that low-volume or focal nodal involvement may contribute to false-negative FNA results through sampling of an uninvolved portion of a metastatic node or a different, noninvolved lymph node. Krishnamurthy et al. further proposed that incomplete sonographic identification of all abnormal lymph nodes may contribute to discrepancies between preoperative FNA and final surgical staging [11], whereas Mainiero et al. emphasized that the most operator-dependent aspect of ultrasound-guided FNA is selecting the most suspicious lymph node for aspiration rather than performing the aspiration itself [13]. Accordingly, negative ALN FNA results, particularly in clinically or radiologically suspicious lymph nodes, should not preclude surgical staging, as also recommended by Sallout et al. [12].

The atypical and suspicious for malignancy categories illustrate additional challenges in lymph node cytology. Although the number of cases was small, both atypical cases and most suspicious cases were malignant on follow-up. Common factors contributing to indeterminate interpretations included scant atypical cells, obscuring blood or fibrin, acellular cell blocks, and negative or equivocal immunohistochemical results. Low-volume nodal disease, including isolated tumor cells, may further complicate cytologic assessment because tumor cells may be extremely scant or may not be sampled by FNA. These findings support repeat sampling, core needle biopsy, or close clinicoradiologic correlation, especially when atypical results are encountered.

Two lobular carcinoma cases with suspicious and atypical cytology diagnoses illustrate the prior concept that the individual cell deposition of lobular carcinoma in cytology represents a diagnostic challenge [30,31]. The recognized cytologic difficulty of invasive lobular carcinoma was reflected in our exploratory subtype analysis. ALN FNA demonstrated lower sensitivity for ILC than IDC (64.3% vs. 89.5%) and a correspondingly higher false-negative rate (35.7% vs. 10.5%; *p* = 0.022). This finding is consistent with the tendency of lobular carcinoma to involve lymph nodes as dispersed individual cells that may be difficult to distinguish from lymphocytes and may be more readily missed by limited FNA sampling. However, because only 14 ILC cases with metastatic disease were evaluable for sensitivity, these findings require validation in larger cohorts.

The malignant category showed excellent predictive value, with only a single discordant case among 123 cases with follow-up. Notably, some malignant cases had no residual nodal metastasis but showed treatment effect in lymph nodes following neoadjuvant chemotherapy, whereas others had biopsy-proven distant metastatic disease. The 99.2% ROM observed in this category supports the high reliability of a malignant ALN FNA diagnosis, while also highlighting the importance of considering treatment-related changes when interpreting subsequent pathologic findings.

An additional consideration is specimen adequacy. Although there is no standardized minimum number of lymphocytes required to classify a lymph node FNA as adequate for cytologic evaluation [14], our findings suggest that adequacy criteria may need to be considered more cautiously in the setting of axillary staging for invasive breast carcinoma. Several false-negative cases showed scant lymphoid cellularity, and half of the non-diagnostic cases with follow-up were positive for metastatic carcinoma. Given the high pretest probability of metastatic disease in patients undergoing ALN FNA for clinically or radiologically suspicious lymph nodes, scant lymphoid cellularity should be interpreted cautiously before a specimen is considered adequate for a benign diagnosis.

More than half of the patients in this cohort received NACT. Because ALN FNA was performed before initiation of systemic therapy, NACT did not directly influence the cytologic findings; however, it affected interpretation of subsequent lymph node pathology used for correlation. In particular, 38 patients with malignant ALN FNA subsequently had negative lymph nodes showing treatment effect, consistent with eradication of previously documented nodal metastasis. These cases were therefore considered concordant with the pretreatment positive FNA rather than false-positive results. Consequently, treatment-related changes in the reference specimen must be considered when assessing the diagnostic performance of pretreatment ALN FNA.

This study has several limitations. First, it was a retrospective single-institution study subject to selection bias. Second, not all cases had surgical follow-up, although clinical follow-up was available for a subset of patients. Third, the non-diagnostic, atypical, and suspicious for malignancy categories contained relatively few cases, resulting in wide 95% confidence intervals and limited precision of the corresponding ROM estimates. Accordingly, these ROM estimates should be considered exploratory and not be directly generalized to other patient populations, particularly settings with a lower pretest probability of metastatic breast carcinoma. Larger multicenter studies are needed to establish more precise and generalizable category-specific ROM estimates. Fourth, WHO categorization was performed retrospectively by a single cytopathologist who was not blinded to the original cytologic diagnosis or subsequent follow-up, and interobserver reproducibility was not assessed. Although knowledge of subsequent outcomes introduced the potential for review bias, no cases were reassigned to a different diagnostic category during retrospective review. Fifth, the use of different reference standards represents an additional limitation. Although most cases had pathologic follow-up, 20 were evaluated by clinical and/or radiologic follow-up alone, and 64 without adequate follow-up were excluded. Because the likelihood and type of pathologic verification may have been influenced by the initial cytologic diagnosis and subsequent treatment, verification bias cannot be excluded and may have affected ROM estimates and measures of diagnostic performance. Finally, variability in ultrasound-guided sampling technique among operators may influence the diagnostic performance of ALN FNA, but operator-related variability was not specifically evaluated.

## 5. Conclusions

In summary, our cases support the assertion that ALN FNA is a highly effective method for the preoperative assessment of ALN involvement in patients with invasive breast carcinoma. The malignant and suspicious categories are highly predictive of metastatic disease, whereas atypical diagnoses warrant additional evaluation because of their substantial ROM. The principal source of false-negative results appears to be sampling limitations rather than interpretive error. However, the 17% ROM observed in the benign category indicates that a negative ALN FNA does not reliably exclude metastatic disease, and surgical nodal staging may still be warranted in clinically or radiologically suspicious cases. These findings support the clinical utility of ALN FNA and provide breast carcinoma-specific ROM estimates that may facilitate application of the WHO System for Reporting Lymph Node Cytopathology in this setting.

## Figures and Tables

**Table 1:** Risk of malignancy and 95% confidence intervals for low-frequency WHO diagnostic categories.

WHO Category	Malignant/Follow-up	ROM	95% CI
Non-diagnostic/insufficient	3/6	50.0%	11.8–88.2%
Atypical	2/2	100%	15.8–100%
Suspicious for malignancy	6/7	85.7%	42.1–99.6%

Category-specific risk of malignancy (ROM) was calculated among cases with adequate follow-up. The wide 95% confidence intervals reflect the small number of cases in the non-diagnostic/insufficient, atypical, and suspicious for malignancy categories and indicate limited precision of these ROM estimates. CI, confidence interval; ROM, risk of malignancy; WHO, World Health Organization.

**Table 2:** Distribution of ALN FNA diagnostic categories according to breast carcinoma histologic subtype.

Histologic Type	*N*	Benign	Atypical	Suspicious	Malignant	Non- Diagnostic
**IDC**	204	69 (33.8%)	1 (0.5%)	6 (2.9%)	124 (60.8%)	4 (2.0%)
**ILC**	23	12 (52.2%)	0	1 (4.3%)	9 (39.1%)	1 (4.3%)
**Mixed ductal/lobular**	20	4 (20.0%)	1 (5.0%)	0	15 (75.0%)	0

Cytologic diagnoses are presented according to the WHO System for Reporting Lymph Node Cytopathology. Values represent the number of cases within each diagnostic category, with percentages calculated within each histologic subtype. ILC was much less often called malignant on FNA than IDC (39.1% vs. 60.8%) and was more often classified as benign (52.2% vs. 33.8%). ALN, axillary lymph node; FNA, fine-needle aspiration; IDC, invasive ductal carcinoma; ILC, invasive lobular carcinoma.

**Table 3:** Sensitivity and false-negative rate of ALN FNA according to breast carcinoma histologic subtype.

Histologic Subtype	True Positive	False Negative	Malignant Cases Evaluable for Sensitivity	Sensitivity	False-Negative Rate
**IDC**	94	11	105	89.5%	10.5%
**ILC**	9	5	14	64.3%	35.7%
**Mixed ductal/lobular**	13	2	15	86.7%	13.3%

Analysis was restricted to cases with metastatic nodal disease and adequate follow-up that were evaluable for calculation of sensitivity. Sensitivity was calculated as true-positive cases divided by the total number of true-positive and false-negative cases; the false-negative rate was calculated as false-negative cases divided by the same denominator. ILC demonstrated lower sensitivity (64.3%) and a higher false-negative rate (35.7%) than IDC (89.5% and 10.5%, respectively) and mixed ductal/lobular carcinoma (86.7% and 13.3%, respectively). ALN, axillary lymph node; FNA, fine-needle aspiration; IDC, invasive ductal carcinoma; ILC, invasive lobular carcinoma.

**Table 4:** Comparison of category-specific risk of malignancy in this study with published WHO estimates.

WHO Diagnostic Category	WHO Reported ROM Range	WHO Pooled ROM	Present-Study ROM	Absolute Difference vs. Pooled WHO ROM
**Non-diagnostic**	17–67%	34%	50.0%	+16.0 percentage points
**Benign**	2–16%	5%	17.0%	+12.0 percentage points
**Atypical**	29–77%	65%	100%	+35.0 percentage points
**Suspicious for malignancy**	88–100%	93%	85.7%	−7.3 percentage points
**Malignant**	98–100%	100%	99.2%	−0.8 percentage points

Published WHO ROM ranges and pooled ROM estimates are shown for each diagnostic category and compared with the corresponding ROM observed in this cohort of patients undergoing ALN FNA for invasive breast carcinoma. The absolute difference represents the present-study ROM minus the pooled WHO ROM and is expressed in percentage points. Differences were greatest for the non-diagnostic, benign, and atypical categories, whereas ROMs for the suspicious for malignancy and malignant categories were similar to published WHO estimates. ALN, axillary lymph node; FNA, fine-needle aspiration; ROM, risk of malignancy; WHO, World Health Organization.

## Data Availability

Dataset available upon request from the authors.
